# Supplementary material for: Temperature dependence of adhesive friction properties of rubber asphalt mortar and its mechanism of action
Source: PLoS One. 2025 May 27;20(5):e0321059. doi: 10.1371/journal.pone.0321059 (PMC12112197; doi:10.1371/journal.pone.0321059)
Supplement: Attachment — Submitted filename: Response to Reviewers.docx [file pone.0321059.s010.docx]

**Response to Reviewers**

Dear Editors and Reviewers:

Thank you for your letter and for the reviewers’ comments concerning our manuscript entitled ' Study on the temperature dependence of adhesive friction properties of rubber asphalt mortar and its mechanism of action' (Manuscript Number: PONE-D-24-53366). Those comments are all valuable and helpful for revising and improving our paper, as well as an important guide for our research. We have studied the comments carefully and made revisions. Revised parts are marked in red on the paper. Our responses to reviewers' comments are summarised below.

I would like to thank the editors and reviewers for their work and thank you for giving us the opportunity to be able to submit a revised version of this paper. Thank you again for your comments and suggestions.

Sincerely yours,

Ning Li

**Response to Reviewers #1:**

**Point 1:** The manuscript presents a good and important qualitative work that attempts to fill the knowledge gap in the partial composition of the asphalt mixture components. It did not present a new proposal and was satisfied with listing the mechanism for applying or using the program.

**Response 1:** Thank you very much for your valuable comments, which have significantly improved the quality of our paper. Regarding your point that the manuscript did not present a new proposal and was only satisfied with listing the mechanism for applying or using the program, I fully understand your concerns. However, the main focus of this paper is to establish a three - layer friction pair model of rubber - asphalt mortar - aggregate, simulate the adhesive friction performance of the interface friction system, analyze the variation laws of the friction performance of rubber asphalt mortar under different temperature conditions and different rubber powder content conditions, and explain the mechanism of the adhesive friction performance of rubber asphalt mortar. Based on the new proposal you mentioned about the temperature - dependence of the adhesive friction performance of rubber asphalt mortar mixtures and its mechanism, it remains an important issue that requires further exploration. In light of your valuable suggestions, we have reviewed a large number of literatures. In the subsequent research, the authors will conduct in - depth analysis and discussion on the new proposal regarding the temperature - dependence of the adhesive friction performance of rubber asphalt mortar and its mechanism. We sincerely appreciate your guidance and will continue to strive to improve the quality of our research.

Once again, the authors would like to thank you for your valuable comments and for reviewing the manuscript!

**Point 2:** The manuscript is written in a format closer to a report or guide to represent the partial components of the asphalt mixture.

**Response 2:** Thank you for your valuable comments, which have significantly enhanced the quality of our paper. Regarding your concern that the writing format of this manuscript is more akin to a report or guide introducing the partial components of the asphalt mixture, I fully understand. In this study, however, the authors utilized Materials Studio 2020 software to construct the molecular model systems of rubber asphalt mortar and the rubber asphalt mortar - aggregate interface under the actual powder - binder ratio conditions. The models were verified, and their physical properties were simulated. Subsequently, on this basis, a three - layer friction pair model of rubber - asphalt mortar - aggregate was established. Finally, the adhesive friction performance of the interface friction system was simulated, the variation laws of the friction performance of rubber asphalt mortar under different temperature conditions and different rubber powder content conditions were analyzed, and the mechanism of the adhesive friction performance of rubber asphalt mortar was explained. We sincerely appreciate your guidance and will continue to strive to improve the quality of our research.

Once again, the authors would like to thank you for your valuable comments and for reviewing the manuscript!

**Point 3:** It is very important that the real practical importance of the aim of the current study.

**Response 3:** Thank you very much for your meticulous guidance on this manuscript. We have carefully considered and analyzed the issues you raised regarding this manuscript. This study is of great significance in practical applications, which is mainly reflected in the following aspects: The adhesive friction performance of rubber asphalt mortar directly affects the skid resistance and durability of roads. By simulating the variation laws of friction performance under different conditions, the research can provide more adaptable materials for road construction in different climate regions, ensuring that roads can maintain good performance under various temperatures. We sincerely appreciate your guidance and will continue to strive to improve the quality of our research.

Once again, the authors would like to thank you for your valuable comments and for reviewing the manuscript!

**Response to Reviewers #2:**

**Point 1:** Title: Change the title to be ((temperature dependence of adhesive friction properties of rubber asphalt mortar and its mechanism of action).

**Response 1:** Thank you very much for your careful guidance on this manuscript. We have carefully considered and analyzed the issues you raised about this manuscript. We agree with your opinion. We have carefully and specifically elaborated in the text and replaced the relevant sections with red markings. Your comments have greatly improved the quality of our manuscript!

**Point 2:** Abstract: Remove the first sentence ((Abstract: Excellent pavement skid resistance guarantees the friction resistance required for braking between tires and roads.))

**Response 2:** Thank you very much for your meticulous guidance on this manuscript. We have carefully considered and analyzed the issues you raised regarding this manuscript. We have deleted the first sentence of the abstract ("Excellent pavement skid resistance guarantees the friction resistance required for braking between tires and roads"). Your comments have significantly enhanced the quality of our manuscript!

**Point 3:** Conclusions rather than ((conclusion)). The conclusions must expose what was found from behind the results; not repeating the results only. Rewrite the conclusions.

**Response 3:** Thank you for your valuable comments, which have significantly enhanced the quality of the paper. In response to your concerns, the authors have carefully re-summarized the conclusions to expose the discoveries behind the research results, rather than merely repeating the results. The revised parts in the paper are marked in red. The specific revised content is as follows:

**Revised version:**

(1) The increase in temperature promotes phase transition and the enhancement of adhesive, which improves the friction performance of rubber asphalt mortar. As the temperature rises, the friction coefficient between rubber asphalt mortar and the rubber plane shows an increasing trend. The degree of fitting between the simulated values and the predicted values is good, with a maximum difference of 0.32.

(2) The addition of rubber powder helps improve the mechanical properties of the mortar. As the rubber powder content and temperature increase, both the bulk modulus and Young's modulus of rubber asphalt mortar show an upward trend.

(3) Based on the simulation and analysis of the three-layer friction molecular dynamics model of rubber-asphalt mortar-aggregate, it can be seen that the addition of rubber powder optimizes the friction performance of the mortar to a certain extent.

(4) Through the analysis of molecular order and density, it has been verified that the molecular models of rubber asphalt mortar constructed under different rubber powder contents (0%, 10%, 20% and 30%) can truly and reliably reflect the physical properties of rubber asphalt mortar.
